# Effects of whole-body electromyostimulation training on upper limb muscles strength and body composition in moderately trained males: A randomized controlled study

**DOI:** 10.3389/fpubh.2022.982062

**Published:** 2022-09-09

**Authors:** Yin Qin, Hui Chen, Xiaoying Liu, Jiwei Wu, Yinxin Zhang

**Affiliations:** ^1^Department of Rehabilitation Medicine, The 900th Hospital of Joint Logistic Support Force, PLA, Fuzhou, China; ^2^Department of Rehabilitation Medicine, Fuzong Clinical Medical College of Fujian Medical University, Fuzhou, China

**Keywords:** electric stimulation, WB-EMS, strength, body composition, moderately trained

## Abstract

Resistance training has been known to have a positive effect on muscle performance in exercisers. Whole-body electromyostimulation (WB-EMS) is advertised as a smooth, time-efficient, and highly individualized resistance training technology. The purpose of this study is to evaluate the effects of WB-EMS training on maximum isometric elbow muscle strength and body composition in moderately trained males in comparison to traditional resistance training. The study was a randomized controlled single-blind trial. Twenty, moderately trained, male participants (25.15 ± 3.84, years) were randomly assigned to the following groups: a WB-EMS training group (*n* = 11) and a traditional resistance training group (the control group [CG]: *n* = 9). Both training intervention programs consisted of 18 training sessions for six consecutive weeks. All subjects performed dynamic movements with the WB-EMS or external weights (CG). The primary outcome variables included maximum isometric elbow flexor strength (MIEFS), maximum isometric elbow extensor strength (MIEES) and surface electromyography amplitude (sEMG_RMS_). Secondary outcomes involved lean body mass, body fat content, arm fat mass, and arm lean mass. ANOVAs, Friedman test and *post hoc t*-tests were used (*P* = 0.05) to analyze the variables development after the 6-week intervention between the groups. Significant time × group interactions for MIEFS (η^2^ = 0.296, *P*_*Bonferroni*_ = 0.013) were observed, the increase in the WB-EMS group were significantly superior to the CG [23.49 ± 6.48% vs. 17.01 ± 4.36%; MD (95% CI) = 6.48 (1.16, 11.80); *d* = 1.173, *P* = 0.020]. There were no significant differences were observed between interventions regarding MIEES, sEMG_RMS_ and body composition. These findings indicate that in moderately trained males the effects of WB-EMS were similar to a traditional resistance training, with the only exception of a significantly greater increase in elbow flexor strength. WB-EMS can be considered as an effective exercise addition for moderately trained males.

## Introduction

Improving muscle strength and body composition are important goals for mastering regular activity and improving physical fitness as well as preventing age-related diseases (1, 2). Resistance training has a favorable effect on these two indicators (3–6) and is recognized as an integral part of exercise programs that improve and maintain physical fitness and health (7). Several studies have demonstrated that cycle training loads of individuals with years of resistance training experience corresponding to 80–100% of 1 repetition maximum (RM) (7), which is effective in maximizing muscle strength and has a beneficial effect on body composition (6, 8). Traditional resistance training is based on the principle of progressive overload and excessive compensation, and typically involves movement with heavy load and of high intensity. During this type of training, the resulting joint moment (torque), shear force, and pressure can be extremely high. Meanwhile, high training volumes are associated with increased injury rate (9). Strain, tendinitis, and sprains are the most common types of injuries (10) and are usually caused by overuse. In addition, resistance training classically uses gravity acting upon resistance, including bodyweight or specialized fitness devices (most commonly barbells, dumbbells, and resistance-training machines) to target muscle groups and joint movements for targeted trainings. However, fitness device exercises have significant time and venue constraints, making it difficult for exercisers with limited time and spatial resources to practice such exercises. Time stress is frequently cited as a major barrier to frequent exercising (11) in both active and sedentary individuals (12, 13). Hence, time-efficient and low-load exercise protocols with portable devices might be better alternatives for exercisers to improve their physical fitness. In recent years, whole-body electromyostimulation (WB-EMS) has been increasingly applied in the field resistance training, as a joint-friendly, time-efficient, and highly individualized supplementary training method.

WB-EMS is a suit-like wearable drive that makes it possible to globally combine electrical muscle stimulation with functional movement exercise programming. Electrical stimulation training induces muscle contraction by stimulating certain muscle nerves with pulsed electric currents at different frequencies, which may improve protein synthesis and muscle mass growth. Previous studies have shown that WB-EMS improves health-related outcomes (14) such as appendicular muscle strength (15–17), sport performance (1, 18) and body composition (15). Compared with traditional techniques, WB-EMS has the advantage of directly acting on the synthesis of skeletal muscle proteins and prioritizing the activation of type II skeletal muscle fibers (16). It was developed to generate adaptability through a nonselective synchronous recruitment of muscle fibers and by increasing impulse firing rate to achieve greater activation of motor units. WB-EMS reduces training volume by intensifying exercises (19). Thus, superimposed WB-EMS can be considered less time-consuming than the already efficient high-intensity training program to improve body composition and strength (20); besides, with WB-EMS, a lower volume of injury incidence is conceivable.

However, there has been a continuous debate about the efficacy of EMS in healthy people with a long sports experience. Some studies have shown that superimposed EMS training increases motor unit recruitment, and thus induces an increased physiological response and consequent adaptation of the skeletal muscle (21–23). Until now, it remains unclear whether EMS training leads to greater neural adaptation (muscle activation) and muscular adaptation (e.g., muscle strength) than pure exercise training (with weight) in trained people. Several recent studies (24) have shown that WB-EMS has limited effect in moderately trained people. Interestingly, most previous studies have focused on lower limbs, and the impact on the upper limb is often ignored. A recent study (25) compared the effects of strength training, EMS, and a combination of both on elbow flexor muscle morphological adaptations in trained healthy young adults. The study reported a significant improvement in this parameter after EMS combined with voluntary contraction training. However, they did not support the idea that superimposed EMS is a better approach, although only flexors' muscle thickness was assessed. Therefore, this study investigated the neuromuscular adaptations of the upper arm flexors and extensors to complement the evidence for the effects of WB-EMS training in trained-state population. In addition, positive effects of WB-EMS on body composition have been reported in untrained population, with considerable heterogeneity in results (15). It has been clearly proven that the energy expenditure of low-intensity resistance training with WB-EMS is higher than that of traditional resistance training (19). However, the small effect is not enough to support WB-EMS as an expensive alternative training technique for “body shaping”. The effect of this training on body mass and fat content remains to be determined, and no studies have reported evidence of an effect on body composition in the upper arms.

Therefore, the purpose of this study is to determine the effects of WB-EMS training program on upper limb muscle strength and body composition in moderately trained males in comparison to traditional resistance training. Specifically, we hypothesized that the WB-EMS associated dynamic movement is more effective in improving muscle strength and body composition than traditional resistance exercises. Such information would allow exercisers and trainers to consider WB-EMS training possibilities to as highly efficient techniques that reduce training volume and time consumption.

## Materials and methods

### Trial design

**Figure 1:**
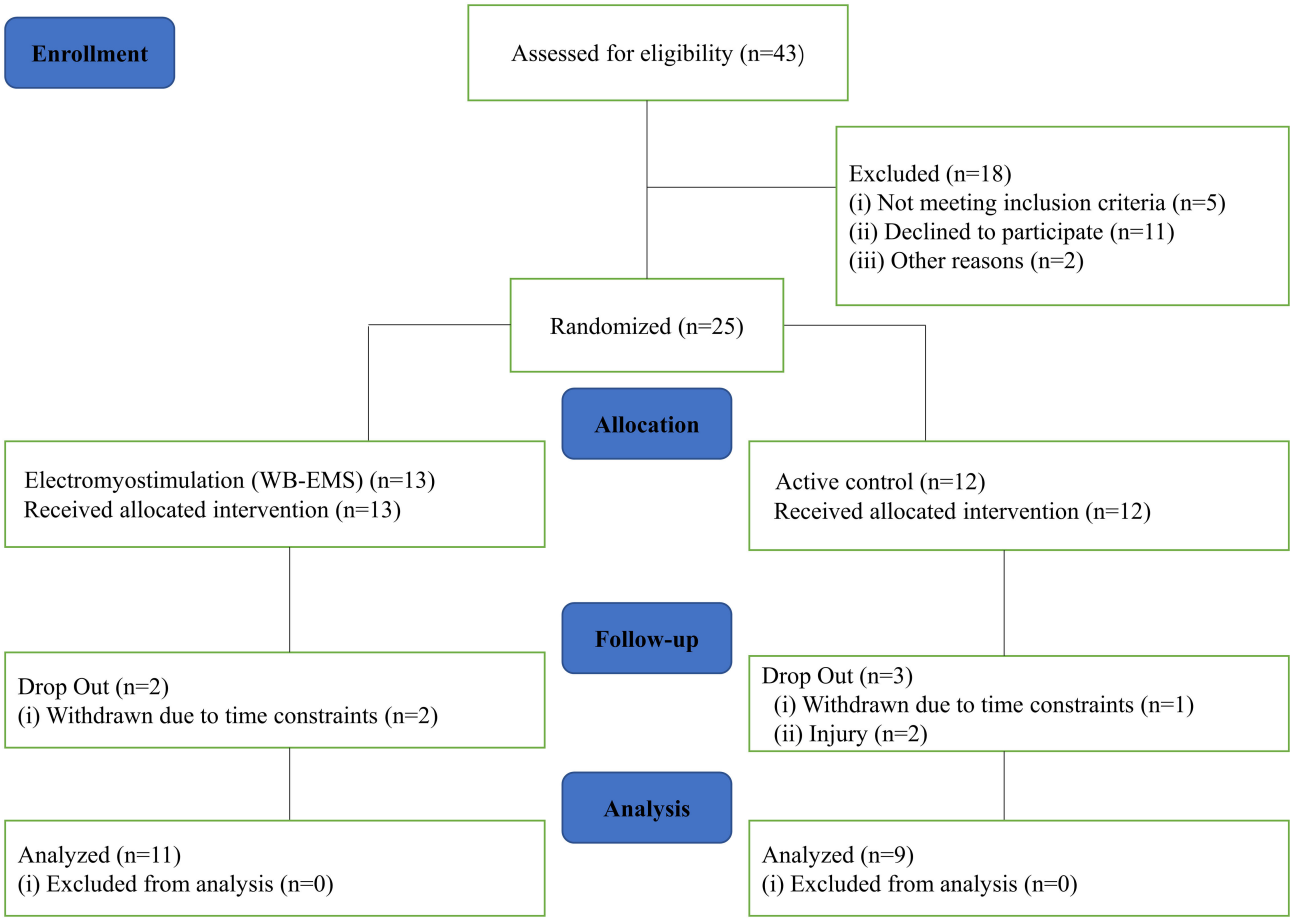
Flowchart of the planned protocol pathway.

The study was a randomized controlled single-blind trial, involving moderately trained males aged 18–40 years. Two groups were randomly formed: a WB-EMS group (where subjects underwent dynamic movements superimposed on WB-EMS) and a control group (CG), where subjects received traditional resistance exercise without WB-EMS. Both training intervention programs consisted of 18 training sessions for six consecutive weeks. Before and after the 6-week training, isometric elbow muscle strength, muscle activation, and body composition were measured to determine the effects of the training. The flowchart of this study is presented in Figure 1. The protocol was registered at the Chinese Clinical Trials Registry (ChiCTR2200060338) and approved by the Research Ethics Committee of the 900th Hospital of the Joint Logistics Support Force of the Chinese People's Liberation Army (No. 2022-13).

The study assessors informed eligible participants about the specificities, benefits, and possible risks (such as radiation from X-rays) of the study. Before being assessed for eligibility, volunteers read and signed an informed consent form after having accepted the invitation to participate in the trial. To minimize the effect of non-specific exercises on the results of the experiment, participants were required to maintain their daily exercise habits and not to do additional upper limb exercises. In addition, the participants were asked to maintain their habitual lifestyle and nutrition. The identity of the participants was protected by a third party (not involved in the research) who omitted their identification information by coding.

#### Participants

The sample size was determined using the G^*^Power3 program. For an effect size of 0.57, the type I error probability of 0.05 and a power of 0.80, a minimum total sample size of 20 participants. Participants were recruited through announcements and social media messages (May and June 2022). Most of the volunteers came from sports colleges or amateur sports clubs. A total of 43 people were registered for the program, and 25 were eventually considered eligible for inclusion in the study. Our inclusion criteria were (a) male, aged 18 to 40 years; (b) >2 years of experience in exercise, 5–8 h of training sessions per week; (c) good health status on physical examination; and (d) no experience of electrical stimulation training. The exclusion criteria were as follows: (a) absolute contraindication for WB-EMS (e.g., epilepsy, metal implants, cardiac pacemakers); (b) muscle, heart, or inflammatory disease; (c) present intake of medications that affect muscle metabolism; (d) sports injury in the past 3 months; (e) surgical operations in the last 6 months; and (f) injury or illness during the program.

### Training protocol

#### The WB-EMS group

In this study, WB- EMS was performed during the eccentric contractions part of movements, returning to the starting position during the rest interval. A bipolar electric current by WB-EMS (PE-FCV-V02, China) was applied with the following parameters: current frequency = 85 Hz, pulse width = 350 μs, current pulse = 6 s and rest interval = 4 s (duty cycle was 3:2). The electrodes of the WB-EMS covered major muscle groups throughout the body, including the chest (pectoralis major, pectoralis minor), abdomen (rectus abdominis), upper back, lower back (latissimus dorsi, erector spinae, iliopsoas), upper limbs (deltoid, biceps, triceps), buttocks (gluteus maximus), and legs (quadriceps, hamstrings). It is usually recommended to wet the training gear with water to ensure optimum electrical impulse transmission to muscles. Current intensity was individually selected and adjusted for the same session. An electrical stimulation adaptation time of 2–3 min was provided before each training session to improve the participants' adaptability and minimize possible experimental damage (26). Incremental exercises were not performed in this study.

Participants underwent 20 min of WB-EMS training (all without additional weight) three times a week for 6-week. The first 10 min of non-specific exercises included squats, chest press, butterfly reverse, and standing diagonal crunches. The last 10 min of the WB-EMS session involved arm training (e.g., biceps curl and arm extension). The muscles were warmed up and relaxed for 10 min before and after each session. Most studies (27, 28) use regulated maximum pulse intensity based on the maximum pain threshold (maximum tolerated amperes) which causes high muscle tension and limits the range of dynamic movements. In this study, participants individually measured the maximum intensity of the acceptable current before the exercise and trained at 80–100% of the individual pain threshold amperage. The modification of the current intensity was based on the completion of dynamic motion. Relative to regular exercise programs, the current intensity was essential to achieve positive results; therefore, current intensity had to be taken with caution. Due to the fact that stimulated sites' impedance differences have many influencing factors (e.g., skin, fat, muscle thickness), the subjects maintained a rate of perceived exertion (RPE) of “Hard (heavy)” to “Very hard” (Borg CR-10 “6” of “10”) during the session to individually adjust exercise intensity.

The session was supervised by a single instructor and two therapists. The instructor was responsible for guiding participants through the program. The therapists monitored the exercise performance and paid attention to the subjects' condition during the session. Following the training, if any rheumatic pain persisted for more than a few hours, the participant's next session was adjusted to be less intensive, as appropriate.

#### The control group

The CG training consisted of the same 10-min warm-up as in the WB-EMS group, including running and movement of the different joints. Resistance training consisted of a 6-week training on fitness devices (squats, chest press, butterfly reverse, standing diagonal crunches, and the arm training). Exercise equipment used includes dumbbells, barbells, elastic bands, butterfly machine, and seated chest press. The participants underwent traditional resistance training thrice per week. The training load ranged from 80 to 100% of one repetition maximum (29), and each movement was repeated 15 times (3 sets × 5 repetitions). The arm training included biceps curl and arm extension, with 30 resistance exercises each (6 sets × 5 repetitions). The duration of each movement in the CG was 2 s eccentric, 1 s isometric distance and 2 s concentric. Rest intervals of at least 5 s between repetitions and 3 min between sets were defined. No incremental load was applied during the training.

### Randomization procedure

A researcher who did not participate in the trial randomly divided eligible participants into two study groups, using an online statistical computing web programming (www.randomization.com). The subjects (twenty-five) were numbered and assigned random numbers according to the order of registration. Ranked the random numbers from big to small, the first 13 into the WB-EMS group. None of the participants or researchers knew about the distribution in advance. After being assigned to one of the groups, the researchers made the subjects understand the procedures in detail.

### Blinding

Due to the nature of this trial, it was impossible to blind the subjects to their exercise status. However, assessments were performed by an assessor who was blinded to the subjects' group allocation.

### Outcomes

The measured primary outcomes included the following: maximal isometric elbow flexor muscle strength (MIEFS), maximal isometric elbow extensor muscle strength (MIEES), and neuromuscular activation recorded using surface electromyography amplitude (sEMG_RMS_) for the biceps brachii and triceps brachii muscles.

Secondary outcomes were: body composition, including lean body mass (LBM), body fat content, arm fat mass, and arm lean mass.

All participants were requested to suspend any vigorous physical exercise and maintain a normal diet for at least 48 h before the assessments.

#### Maximal voluntary isometric contraction

All force measurements were performed using a handheld dynamometer of brand MicroFET2 (Hoggan Health Industries, West Jordan, UT, USA). Subjects were asked to lie in a supine position with the right elbow flexed at 90°. The dynamometer was clung to the wrist, and the participants' forearms were in supination when measuring the MIEFS, and in forearm neutral position when measuring the MIEES. The participants were required to maintain isometric contractions and perform their best to combat resistance. To avoid compensatory movements that deviated from the measurement results, the assessor familiarized the subjects with the testing process and performed measurements according to the standard. The measurements were performed three times, and the average value was calculated and recorded in Newtons.

#### Surface electromyography (sEMG)

Surface electromyography amplitude of the right elbow flexor and extensor muscles were recorded during maximum voluntary isometric contraction using a surface electromyography apparatus (MyoMove-EOW; Shanghai Ncc Electronic Co., Ltd., Shanghai, China). The skin was cleaned with fine sandpaper and 75% medical alcohol to obtain a low impedance at the skin-electrode interface (*Z* < 1 kΩ). The electrode plates were applied at the thickest part of the length of the biceps brachii and on the lateral head of the triceps brachii muscles; the distance between the plates was 20 mm. The positions of the electrode plates were measured and recorded to reduce errors. The sEMG signals were amplified (500×) and sampled at 2048 Hz prior to being bass-pass filtered in directions between 5 and 500 Hz. The subjects were asked to relax the muscle as much as possible to maintain the sEMG signal near the baseline level before activity. In the process of data acquisition, the participants were asked to do their best to flex/extend the elbow, maintain it for ~5 s, relax for 5 s, and repeat it for three rounds. Using a matching software installed on the surface electromyography apparatus, root mean square (RMS) values were recorded and automatically generated. The RMS of the sEMG signal normalized to isometric peak electromyography was used to assess upper limb muscle activation.

#### Body composition

Whole-body or local body composition was measured by dual-energy X-ray absorptiometry (QDR 4500 A, Hologic, USA), using the instrument's default standard protocol. Subjects were informed of possible radiation problems before measurement, and all metal items were removed prior to each scan. LBM, fat content, arm lean mass, and arm fat mass were record. All the scan information was analyzed independently by a single researcher.

### Statistical analysis

Data were evaluated using the software Statistical Packages for the Social Sciences, SPSS version 26 (IBM, Armonk, NY, USA). The measured data were all expressed as mean ± standard deviation. The statistical significance level was set at *P* < 0.05. Before the statistical analysis, all data were statistically and graphically tested using the Shapiro-Wilk test to verify that the data were normal distribution. The WB-EMS and control groups' all baseline data were analyzed for significant differences by *t*-tests. The changes over time within groups were analyzed by paired *t*-tests. Post-test *the whole-body fat content* (CG: *P* = 0.011) and *arm lean mass* (WB-EMS: *P* = 0.019) parameters were not normally distributed and were analyzed with rank-sum test.

Homogeneity of all parameter variance determined by Levene test. For all variables, variance homogeneity existed and therefore, repeated 2 × 2 ANOVAs (time × group) were calculated based on the raw values for all parameters except *the whole-body fat content* and *arm lean mass*. We used Bonferroni–Holm method for adjusting multiple testing. A test of homogeneity of variances was adopted based on the median scores, where *P* > 0.05 represented equal variances, and the effect size was estimated using Eta squared (η^2^). ES < 0.06 indicated small, 0.06 to 0.14 medium, and > 0.14 strong effects.

To represent differences between the training groups in the pre-post comparison, the variables changes percentage was calculated additionally (Δ% Post-Pre), and then checked after Bonferroni-Holm correction for significant mean differences between the WB-EMS and the CG by means of multiple *t*-tests.

## Results

Forty-three people were initially screened; but after removing non-eligible participants, 25 randomly assigned participants were included in this study. Five subjects (WB-EMS group: *n* = 2; CG: *n* = 3) did not complete the intervention for personal reasons (Figure 1). Finally, 20 participants were included in the per-protocol analysis (Table 1): the CG (*n* = 9, age = 26.89 ± 4.37 years, height = 173.78 ± 2.64 cm, weight = 70.21 ± 2.47 kg, BMI = 23.25 ± 0.77 kg/m^2^, exercise experience = 5.11 ± 2.32 years) and the WB-EMS group (*n* = 11, age = 23.73 ± 2.80 years, height = 174.45 ± 1.75 cm, weight = 73.05 ± 3.77 kg, BMI = 24.01 ± 1.31 kg/m^2^, exercise experience = 4.18 ± 1.99 years). Randomization was considered effective. The baseline parameters of the two groups were not significantly different (all *P* > 0.05), which could have influenced the experimental results.

**Table 1:** Baseline characteristics of experimental subjects.

**Variables**	**WB-EMS**	**CG**	***P*-value**
	**(*n* = 11)**	**(*n* = 9)**	
Age, years	23.73 ± 2.80	26.89 ± 4.37	0.065
Height, cm	174.45 ± 1.75	173.78 ± 2.64	0.500
Weight, kg	73.05 ± 3.77	70.21 ± 2.47	0.068
BMI, kg/m^2^	24.01 ± 1.31	23.25 ± 0.77	0.145
Exercise experience, years	4.18 ± 1.99	5.11 ± 2.32	0.347

Data are presented as the Mean ± SD. WB-EMS, WB-EMS group; CG, control group; BMI, Body Mass Index.

ANOVA showed that none of the parameters exhibited any statistically significant interindividual effect, but had significant intraindividual effects on factor *time* (for MIEFS, MIEES, Biceps Brachii sEMG_RMS_; Triceps Brachii sEMG_RMS_) and *time* × *group* interactions (for MIEFS) with medium high effect sizes (Table 2).

**Table 2:** Result of the 2 × 2-ANOVAs (inter- and intra-individual effects).

	**Inter- individual effects**			**Intra-individual effects**		
	**Group *F*; *p***	** $\eta_{\text{p}}^{2}$ **	**Time *F*; *p***	** $\eta_{\text{p}}^{2}$ **	**Time × Group *F*; *p***	** $\eta_{\text{p}}^{2}$ **
MIEFS	1.56; 0.227	0.080	226.78; <0.001^*^	0.926	7.57; 0.013^*^	0.296
MIEES	0.002; 0.967	0.000	27.23; <0.001^*^	0.602	3.90; 0.064	0.178
Biceps Brachii sEMG_RMS_	0.18; 0.675	0.010	29.43; <0.001^*^	0.620	0.96; 0.341	0.050
Triceps Brachii sEMG_RMS_	0.41; 0.529	0.022	15.18; 0.001^*^	0.457	0.82; 0.378	0.043
Lean Body Mass	3.73; 0.069	0.172	0.24; 0.628	0.013	0.04; 0.849	0.002
Total Body Fat	Not applicable (see text)					
Arm Lean Mass	Not applicable (see text)					
Arm Fat Mass	0.30; 0.590	0.016	1.97; 0.178	0.099	2.48; 0.133	0.121

Reported are *F*- and *p*-values;

* mark significant results at *p* < 0.05; $\eta_{p}^{2}$, effect size partial Eta square.

The Friedman test for *the whole-body fat content* showed no statistically significant difference in the Pre-Post comparison for the WB-EMS (Z = 2.273, *P* = 0.132) and the CG (Z = 0.500, *P* = 0.480), as well as for *arm lean mass* (WB-EMS: Z = 0.091, *P* = 0.763; CG: Z = 1.000, *P* = 0.317).

After the 6-week intervention, MIEFS increased favorably in the WB-EMS group (*P* < 0.001). A significant increase was also observed in the CG (*P* < 0.001). However, the increase in MIEFS was significantly greater (23.49 ± 6.48% vs. 17.01 ± 4.36%; *d* = 1.173; MD [95% CI] = 6.48 [1.16 to 11.80]; *P* = 0.020; Table 3) in the WB-EMS group than in the CG. Likewise, MIEES increased significantly in both groups (WB-EMS: *P* = 0.002; CG: *P* = 0.003) with significant differences between the two groups (18.67 ± 13.41% vs. 8.16 ± 5.47%; *d* = 1.026; MD [95% CI] = 10.52 [0.47 to 20.56]; *P* = 0.041; Table 3).

**Table 3:** Baseline, follow-up data and percentage increases (Δ%) of primary outcomes for the WB-EMS group and the CG.

	**WB-EMS** **(*n* = 11)**	**CG** **(*n* = 9)**	**Difference MV** **(95% CI)**	***P-*value**	**Cohen's d**
MIEFS (N)					
Pre	256.25 ± 25.41	243.44 ± 46.72	–	0.444	–
Post	316.04 ± 30.19	284.76 ± 55.31	–	–	–
Δ% Post–Pre	23.49 ± 6.48	17.01 ± 4.36	6.48 (1.16 to 11.80)	0.020^*^	1.173
MIEES (N)					
Pre	173.96 ± 33.78	183.23 ± 25.12	–	0.504	–
Post	205.56 ± 42.43	197.48 ± 22.99	–	–	–
Δ% Post–Pre	18.67 ± 13.41	8.16 ± 5.47	10.52 (0.47 to 20.56)	0.041^*^	1.026
Biceps Brachii sEMG_RMS_ (μV)					
Pre	806.79 ± 184.98	886.44 ± 301.55	–	0.477	–
Post	1059.32 ± 168.37	1061.88 ± 268.80	–	–	–
Δ% Post–Pre	34.69 ± 23.66	28.15 ± 33.42	6.54 (- 20.29 to 33.37)	0.615	0.226
Triceps Brachii sEMG_RMS_ (μV)					
Pre	486.67 ± 161.69	556.15 ± 182.40	–	0.379	–
Post	611.69 ± 179.33	634.09 ± 152.03	–	–	–
Δ% Post–Pre	29.42 ± 27.17	17.95 ± 20.91	11.48 (– 11.75 to 34.68)	0.313	0.473

WB-EMS, WB-EMS group; CG, control group; Cohen's d, effect size; MIEFS, maximal isometric elbow flexor muscle strength; MIEES, maximal isometric elbow extension muscle strength.

* Mark significant results at *P* < 0.05.

sEMG_RMS_ of the biceps brachii was significantly enhanced in both the WB-EMS (*P* = 0.001) and CG (*P* = 0.007) after the interventions. Significantly increments (*P* = 0.018) were also observed in the WB-EMS group for the triceps brachii twitch contractile properties after the intervention, but not in the CG (*P* = 0.061). However, the change rates in sEMG_RMS_ were not statistically different between the groups (biceps brachii: 34.69 ± 23.66% vs. 28.15 ± 33.42%, *d* = 0.226, MD [95% CI] = 6.54 [– 20.29 to 33.37], *P* = 0.615; triceps brachii: 29.42 ± 27.17% vs. 17.95 ± 20.91%, *d* = 0.473, MD [95% CI] = 11.48 [– 11.75 to 34.68], *P* = 0.313; Table 3).

The LBM changed slightly (WB-EMS: *P* = 0.830, CG: *P* = 0.651) and were not different between the two groups (0.22 ± 2.60% vs. 0.35 ± 2.62%; *d* = 0.050; MD [95% CI] = – 0.13 [– 2.59 to 2.33]; *P* = 0.910; Table 4). The whole-body fat content changes did not differ significantly (3.56 ± 6.57% vs. – 1.56 ± 4.05%; *d* = 0.938; MD [95% CI] = 5.12 [– 0.16 to 10.40]; *P* = 0.057; Table 4). Neither the arm lean mass nor arm fat mass differed significantly from baseline in both groups (WB-EMS group: *P* ≥ 0.722; CG: *P* ≥ 0.061). Similarly, there were no significant differences between the groups in terms of these parameters (arm lean mass: – 0.21 ± 4.30% vs. 1.26 ± 5.82%, *d* = 0.287, MD [95% CI] = – 1.48 [– 6.23 to 3.27], *P* = 0.522; arm fat mass: 1.99 ± 12.48% vs. – 6.47 ± 8.86%, *d* = 0.782, MD [95% CI] = 8.46 [– 1.95 to 18.86], *P* = 0.105; Table 4).

**Table 4:** Baseline, follow-up data and percentage increases (Δ%) of secondary outcomes for WB-EMS group and the CG.

	**WB-EMS** **(*n* = 11)**	**CG** **(*n* = 9)**	**Difference MV** **(95%CI)**	***P-*value**	**Cohen's d**
Lean Body Mass (kg)					
Pre	55.23 ± 3.77	52.35 ± 2.24	–	0.059	–
Post	55.32 ± 3.62	52.56 ± 3.22	–	–	–
Δ% Post–Pre	0.22 ± 2.60	0.35 ± 2.62	– 0.13 (– 2.59 to 2.33)	0.910	0.050
Total Body Fat (%)					
Pre	18.70 ± 3.41	20.28 ± 2.26	–	0.250	–
Post	19.35 ± 3.66	19.99 ± 2.51	–	–	–
Δ% Post–Pre	3.56 ± 6.57	– 1.56 ± 4.05	5.12 (– 0.16 to10.40)	0.057	0.938
Arm Lean Mass (kg)					
Pre	3.36 ± 0.47	3.00 ± 0.36	–	0.072	–
Post	3.34 ± 0.39	3.03 ± 0.39	–	–	–
Δ% Post–Pre	– 0.21 ± 4.30	1.26 ± 5.82	– 1.48 (– 6.23 to 3.27)	0.522	0.287
Arm Fat Mass (kg)					
Pre	0.86 ± 0.24	0.94 ± 0.15	–	0.392	–
Post	0.86 ± 0.21	0.87 ± 0.15	–	–	–
Δ% Post–Pre	1.99 ± 12.48	– 6.47 ± 8.86	8.46 (– 1.95 to 18.86)	0.105	0.782

WB-EMS, WB-EMS group; CG, control group; Cohen's d, effect size.

We had to reject our hypothesis that WB-EMS improved muscle strength and body composition significantly better than traditional resistance training.

## Discussion

The present study aimed to examine the effects of WB-EMS training program on upper limb strength and body composition in moderately trained males in comparison to traditional resistance training. After the 6-week training, statistically significant increases were observed in elbow muscles strength and surface electromyography amplitude in the WB-EMS group. Both types of training had the same effect on body composition; that is, neither training protocol had a significant effect on body composition. Furthermore, WB-EMS training was significantly superior to resistance training in terms of improving elbow flexor muscle strength. The increase in other variables was not restricted to specific training methods, although extensor muscle strength growth rate was higher in the WB-EMS group than in the CG. During the different training sessions, compliance with the training protocol was high (close to 100%) in both groups, with the dropout rate was 20%. We did not observe any significant adverse events in the WB-EMS group. However, in the CG, two participants had to interrupt the training because they incurred injuries; nonetheless, none of the injuries was directly caused by the resistance training protocol. Therefore, we believe that WB-EMS can be considered a safe and effective option to HIT-resistance exercise for people aiming at improving muscle strength and keeping fit.

Our results provide evidence that WB-EMS is effective in improving upper limb muscle strength in moderately trained males. This is important for raising awareness about upper limb development; such as in people who have to bend over their desk while working for long periods of time. Upper limb muscle strength increments identified in the WB-EMS group were between 18.67 and 23.49%. The CG also experienced improved strength (8.16 to 17.01%), but the increase was significantly lower than those in the WB-EMS group. In both groups, decreased strength from the initial value was observed in few individual subjects. This could be due to accumulated fatigue during the 6-week of training or measurement errors of the handheld dynamometer, although prudent measures had been taken to minimize them.

Micke et al. (30) showed that superimposed WB-EMS training at 70% of the maximum tolerated amperage in male sports students, twice a week, produced limited additional effects on strength and power after 8-week, with the only exception being that it induced a significantly greater increase in leg extensor maximal strength. Remarkably, they combined electrical muscle stimulation during squats and glute-ham, which are often used for eccentric training in leg muscles. Eccentric movement, which leads to a lower metabolic and cardiopulmonary demands for a matched workload compared to other contraction modalities (31), is known to be a powerful stimulus for strength growth (32), especially in experienced exercisers (33). In the present study, moderate-intensity (Borg CR-10 “6” of “10”) eccentric resistance training was carried out by superimposed WB-EMS (80–100% of the individual pain threshold amperage). The study by Nicolas Wirtz et al. (34), which showed the potential of EMS on unloaded (antagonistic) muscle groups, supports our results. In von Stengel and Kemmler's study (35) involving non-athletic men (aged 20–35 years), leg flexor and extensor strength increased from 14.7 to 23.2%. These results were similar to the increase rates observed in the present study. Nevertheless, the study by Oliver et al. (17) reported that young (15 to 17 years) elite soccer players showed greater significant gains in strength parameter of ~20.68% (knee flexors) and 33.72% (trunk flexors) after 10-week superimposed WB-EMS training. The reason why the strength gains (18.67–23.49%) in the present study were inferior to those in Ludwig's study might be the facts that in our study, the training period was only 6-week long and no additional weight was applied to all movements. We believe that our moderate-intensity training program ensures a sufficient intensity of exercise stimulation and a lower volume of injury. In addition, the active state of the participants (athletes aged 15 to 17) might also have impacted the results, and WB-EMS might have a more significant effect in younger populations.

EMS activates a higher-threshold motor unit to change at the peripheral level by adapting type II fibers preferentially (16). Studies have shown that superimposed EMS improves the innervation of motor nerves in the body, especially the coordination of agonist and antagonist muscles (36, 37). It may increase the recruitment of motor units, thereby enhancing the physiological response of skeletal muscles and neuromuscular adaptability (21). Through the assessment of neural adaptation (sEMG), this study reported that sEMG_RMS_ signals were significantly changed in the WB-EMS group; but the increase was not significantly different (better) from the increase in the CG. The heterogeneity of the results is probably because most muscle fibers in participants with longer training experience are activated by voluntary contraction, meanwhile the supplementary recruitment of muscle fibers by superimposed WB-EMS is limited to the production of greater neural adaptations (38). On the other hand, early enhancement of maximal voluntary contraction may be caused by structural changes in the central nervous system (39), while the hypertrophic effect is related to changes in muscle strength after a longer stimulation period (less than 6-week) (40, 41). EMS effectively increases the regenerative capacity of satellite cells (42–44), which may induce the proliferation of nuclei through fiber splitting (43, 45). The ability of satellite cells to regenerate increased with the increase in muscle strength and activity of the subjects (42). In an upper-extremity study (25) of people with trained status, low intensity (60% of 10 RM). EMS combined with a strength training protocol significantly increased elbow flexor muscle thickness, showing a positive hypertrophic effect. In a study on elite footballers (16), superimposed WB-EMS was found to significantly augment strength and type II myofiber growth compared to traditional resistance training. Colson et al. (46) reported a significant increase in elbow flexor isometric torque and mechanical twitch after a 7-week EMS training, and that strength gains were not regulated by neural adaptation. In addition, the additional recruitment of muscle fibers induced by WB-EMS may maximize the stimulation of energy expenditure and metabolic stress. In this context, metabolic stress has been recognized as a factor that induces an increase in muscle cross-sectional area. Thus, considering that our study employed high-intensity slow eccentric movement (47–49) combined with WB-EMS, it may be possible to speculate that an additional increase in muscle strength is associated with muscle hypertrophy (50). Moreover, EMS training induced increased cytoplasmic calcium ion concentrations and increased gene expression of myogenic transcription factors D and G in myogenic precursor cells (18). After a single EMS session (42), it has been reported that EMS reduces reactive oxygen species production and improves the global skeletal muscle protein synthesis rates already. Based on the reasons listed above, combined eccentric contraction with WB-EMS may be a better strength-training method for people with moderate training intensity. However, this remains to be verified in this population using a larger sample size.

Based on the discovery that a single WB-EMS session may boost resting metabolic rate and consequently fat metabolism for several hours, at least in healthy persons, it is generally believed that WB-EMS has considerable benefits on fat reduction (20, 51, 52). Our results showed that WB-EMS training generally maintains LBM and has a slight effect on fat. This study used dual-energy X-ray absorptiometry, which is the accepted gold standard for determining LBM. Therefore, we believe that the bias generated by the assessment tool was minimal. The subjects of this study were long-term trainers and were predominantly of normal weight, which may be the reason for the negative results. Previous studies have shown that WB-EMS is effective in improving body composition in sedentary individuals (53), the elderly (54) and sarcopenia patients (52). A study involving healthy male adults reported a significant positive effect of WB-EMS training on lean body mass only; there was no significant reduction in body fat content, even in the overweight cohort (55). However, it is difficult to accurately compare these studies because of the specificities outside the exercise protocol (e.g., age, status, nutritional intake, and means of assessment). In line with the results of this study, another study reported minimal effects of 6-week WB-EMS training on LBM and body mass in athletes (34). Similarly, electrical muscle stimulation devices had slight effects on body weight and body fat in college-aged volunteers in a different report (56). Cohort characteristics have a significant impact on the effectiveness of WB-EMS, with considerable heterogeneity among the results (15).

The study assessors familiarized the participants with the experimental procedures prior to data collection to maximize the test-retest repeatability of the measurements used to assess neural adaptation and strength. All measurements were performed by the same assessor, who was blinded to the identities of the subjects' group allocation. Thus, measurement error and learning effect had little effect on the experimental results. To avoid muscle damage and associated rhabdomyolysis resulting from excessive initial WB-EMS (57), we strictly followed the established training guidelines (26), ruled out severe contraindications, and closely monitored the status of the subjects. In the course of the experiment, no injuries or prolonged discomfort could be referred to the WB-EMS; consequently, the reported protocol could be considered safe for moderately trained men.

Several limitations were found in the current study. First, the trial was carried out with a small sample size, and this may have slightly impaired our results. Further research should therefore be conducted with a larger sample size. Second, the results of the assessment may be skewed by human manipulation or learning effects. Although many steps have been taken to standardize the assessment process as much as possible, there are limitations. In addition, standardized interventions for nutritional intake have not yet been designed. Although we required the subjects to maintain their routine dietary intake habit during the session, we did not take good measures to monitor it. Studies have shown that protein intake during strength training can affect muscle strength and body composition (58, 59). Moreover, the lack of tracking data after detraining hinders the recognition of the delay effect (60, 61).

## Conclusion

The described whole-body electromyostimulation training protocol improves the upper limb strength in moderately trained men to a greater extent than similar traditional resistance training. However, it does not have a significant effect on body composition. Thus, our study supports that whole-body electromyostimulation could be a promising training option to time-efficiently improve muscle strength. Further research should address the observed physiological mechanisms underlying the effect, particularly neuromuscular adaptability.

## Data availability statement

The original contributions presented in the study are included in the article/Supplementary material, further inquiries can be directed to the corresponding author.

## Ethics statement

The studies involving human participants were reviewed and approved by the Research Ethics Committee of the 900th Hospital of the Joint Logistics Support Force of the Chinese People's Liberation Army. The patients/participants provided their written informed consent to participate in this study. Written informed consent was obtained from the individual(s) for the publication of any potentially identifiable images or data included in this article.

## Author contributions

YQ, HC, and XL designed the study. HC, JW, and YZ directed or supervised training. XL evaluated experimental outcomes. HC and YQ completed data analysis, interpretation, and drafted the manuscript. YQ accepts responsibility for the integrity of the data sampling, analysis, and interpretation. All authors contributed to the conception and design of the study, and revised the manuscript accordingly.

## Funding

This study was funded by the Logistical Support Department under the Central Military Commission as the major funding body (CLB19C039).

## Conflict of interest

The authors declare that the research was conducted in the absence of any commercial or financial relationships that could be construed as a potential conflict of interest.

## Publisher's note

All claims expressed in this article are solely those of the authors and do not necessarily represent those of their affiliated organizations, or those of the publisher, the editors and the reviewers. Any product that may be evaluated in this article, or claim that may be made by its manufacturer, is not guaranteed or endorsed by the publisher.
