# Supplementary material for: Effects of whole-body electromyostimulation training on upper limb muscles strength and body composition in moderately trained males: A randomized controlled study
Source: Front Public Health. 2022 Sep 9;10:982062. doi: 10.3389/fpubh.2022.982062 (PMC9501974; doi:10.3389/fpubh.2022.982062)
Supplement: Supplementary file 1 [file Data_Sheet_1.docx]

1. Exercise procedures

| Exercise movements |  |
| --- | --- |
| (1) squats: squats (6s down) and vertical chest press  (2) chest press: squats (6s down) with arm-curls (ext.); return to upright position | |
| (3) butterfly reverse: 6s back | |
| (4) standing diagonal crunches: roll up the upper body (6s down) and bring opposite elbow and knee together. Change sides after each repetition.  (5) arm training: biceps curl and arm extension two sets of each | |

WB- EMS was performed during the eccentric contractions part of movements, returning to the starting position during the rest interval.

2. Whole body electrostimulation protocol design.

| Program variables | Stimulation | |
| --- | --- | --- |
| Stimulation frequency | | 85 Hz |
| Impulse duration | | 6 s |
| Impulse break | | 4 s |
| Pulse breadth | | 350 µs |
| Impulse type | | Bipolar |
| Duration | | ∼20 min |
|  | |  |
